# Supplementary material for: Microbiota and Resistome Analysis of Colostrum and Milk from Dairy Cows Treated with and without Dry Cow Therapies
Source: Antibiotics (Basel). 2023 Aug 14;12(8):1315. doi: 10.3390/antibiotics12081315 (PMC10451192; doi:10.3390/antibiotics12081315)
Supplement: Supplementary file 1 [file antibiotics-12-01315-s001.zip › antibiotics-2500820-supplementary.pdf]

Supplementary material

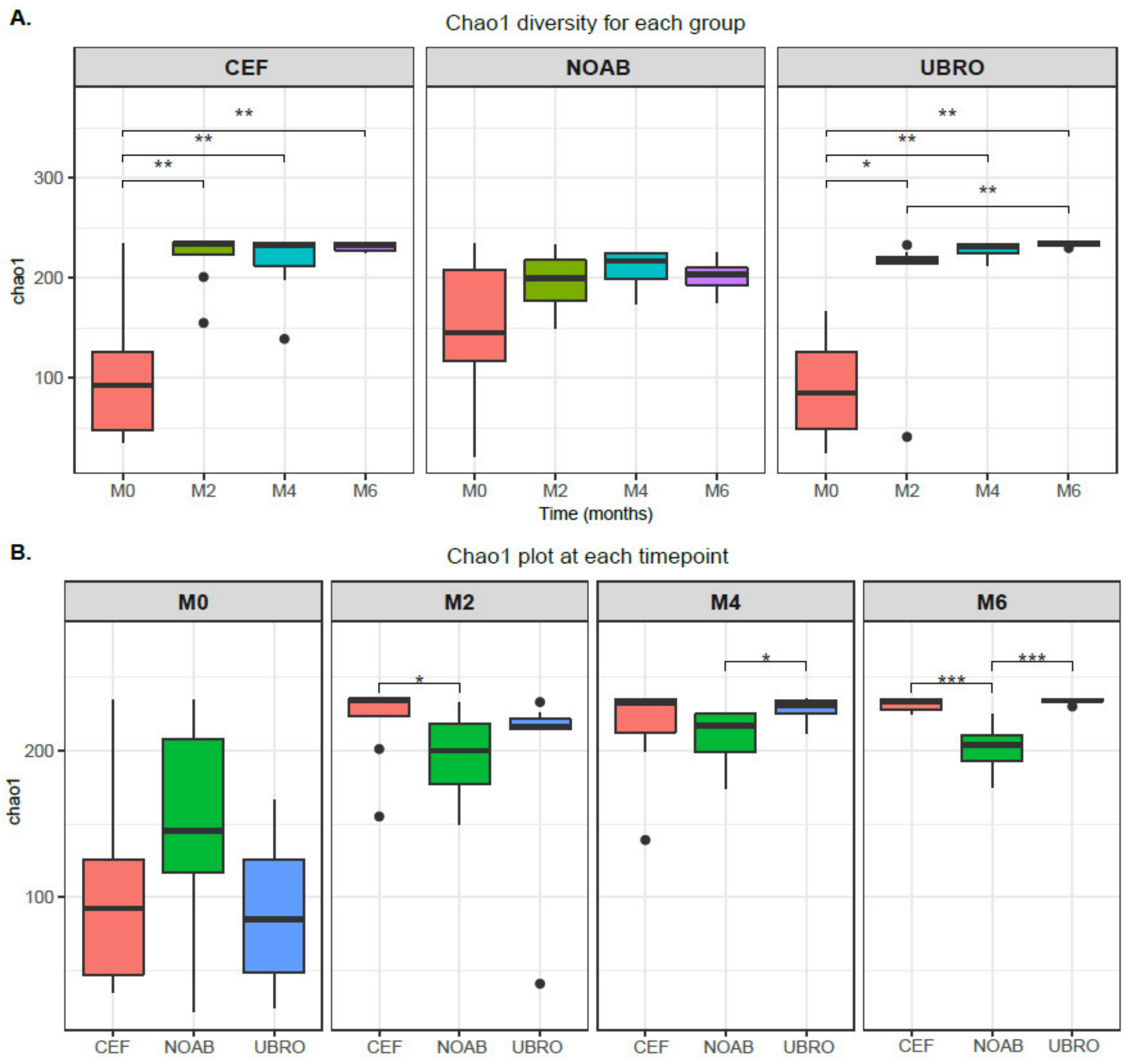

Figure S1. Alpha diversity for microbial composition as calculated by Chao1 index (**A**) Between groups and (**B**) Between time points.

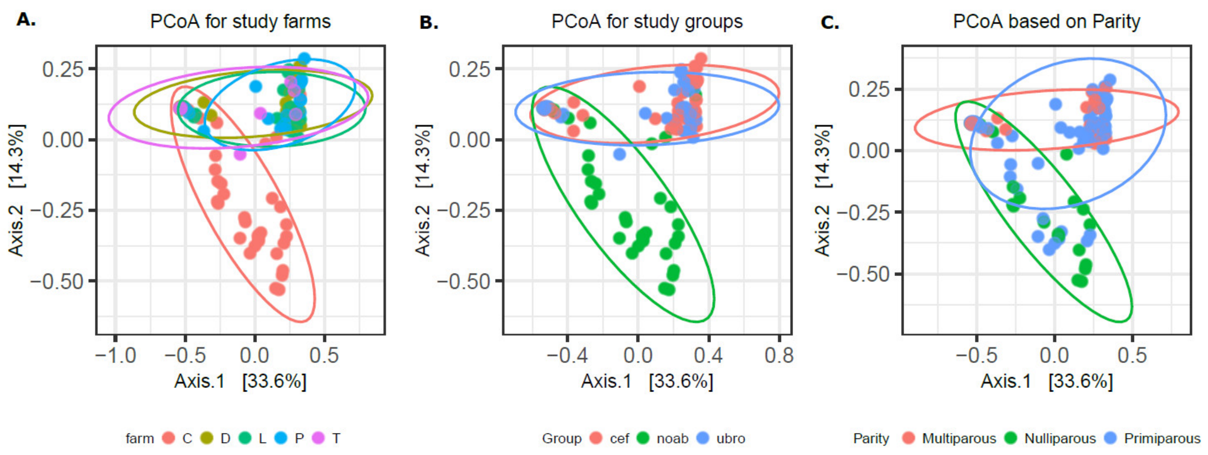

Figure S2. PCoA using Bray-Curtis dissimilarity measure showing distinct clustering based on (**A**) Farms, (**B**) Study groups and (**C**) Parity.

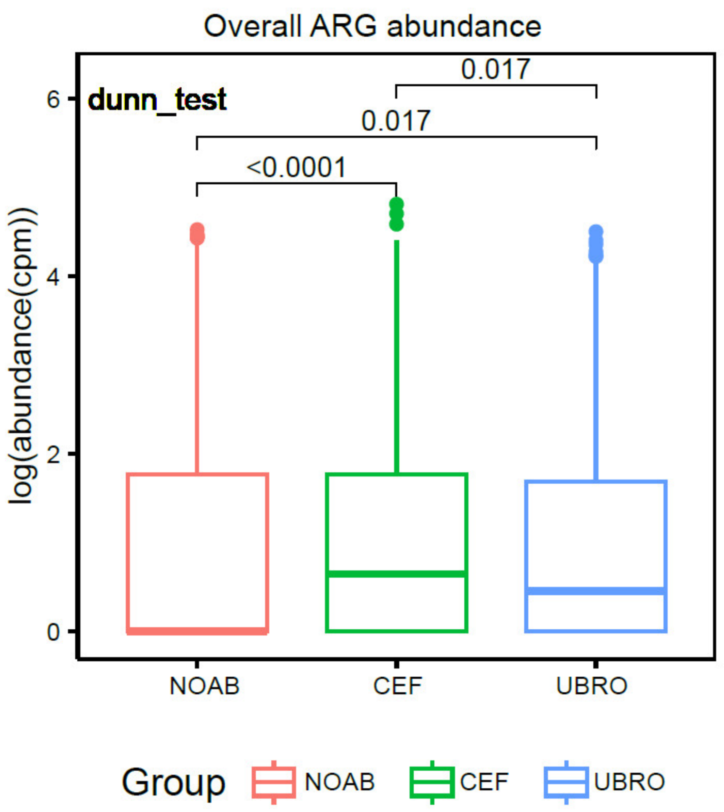

Figure S3. Boxplot showing overall ARG abundance in all three groups. P.adj values were calculated using Dunn test in R, and p.adj values below 0.05 were considered significant.

Table S1. Table showing PERMANOVA results for tests based on farms, study groups and parity.

pairs	R2	p.adjusted
**Farms**		
C vs D	0.11386	0.006667
C vs L	0.119911	0.005
C vs P	0.147541	0.005
C vs T	0.067837	0.036
D vs L	0.044919	0.2025
D vs P	0.085468	0.036
D vs T	0.051612	0.322222
L vs P	0.061289	0.058333
L vs T	0.0343	0.433
P vs T	0.07249	0.16
**Groups**		
noab vs cef	0.119442	0.0015
noab vs ubro	0.109254	0.0015
cef vs ubro	0.026913	0.15
**Parity**		
Primiparous vs Nulliparous	0.05982	0.003
Primiparous vs Multiparous	0.012442	0.418
Nulliparous vs Multiparous	0.114919	0.0045

Table S2. Metadata showing details of all cows included in this study.

Clade	Group	Age	Timepoint	Parity	Parous	Subject
D16T1	cef	4	T1	2	Multiparous	D16
L10C	ubro	5	T0	3	Multiparous	L10
T8T3	ubro	5	T3	3	Multiparous	T8
P10T2	cef	3	T2	1	Primiparous	P10
T17T3	ubro	3	T3	1	Primiparous	T17
T17C	ubro	3	T0	1	Primiparous	T17
C16T1	noab	2	T1	0	Nulliparous	C16
C16C	noab	2	T0	0	Nulliparous	C16
D13C	cef	3	T0	1	Primiparous	D13
C12T1	noab	3	T1	1	Primiparous	C12
D16T3	cef	4	T3	2	Multiparous	D16
C19C	noab	2	T0	0	Nulliparous	C19
L6T1	ubro	3	T1	1	Primiparous	L6
P10C	cef	3	T0	1	Primiparous	P10
L11C	ubro	5	T0	3	Multiparous	L11
L5C	ubro	3	T0	1	Primiparous	L5
C12T3	noab	3	T3	1	Primiparous	C12
C16T3	noab	2	T3	0	Nulliparous	C16
C1T2	noab	3	T2	1	Primiparous	C1
L6T2	ubro	3	T2	1	Primiparous	L6
C16T2	noab	2	T2	0	Nulliparous	C16
C3T1	noab	3	T1	1	Primiparous	C3

P9T1	cef	3	T1	1	Primiparous	P9
L10T1	ubro	5	T1	3	Multiparous	L10
L6T3	ubro	3	T3	1	Primiparous	L6
C5T1	noab	2	T1	0	Nulliparous	C5
C5T3	noab	2	T3	0	Nulliparous	C5
P9C	cef	3	T0	1	Primiparous	P9
D17T3	cef	4	T3	2	Multiparous	D17
P10T3	cef	3	T3	1	Primiparous	P10
C4T3	noab	2	T3	0	Nulliparous	C4
L13T2	ubro	3	T2	1	Primiparous	L13
P20T1	cef	3	T1	1	Primiparous	P20
L5T1	ubro	3	T1	1	Primiparous	L5
D17C	cef	4	T0	2	Multiparous	D17
L11T1	ubro	5	T1	3	Multiparous	L11
C2T1	noab	2	T1	0	Nulliparous	C2
C12C	noab	3	T0	1	Primiparous	C12
C7T3	noab	3	T3	1	Primiparous	C7
L11T3	ubro	5	T3	3	Multiparous	L11
T8T1	ubro	5	T1	3	Multiparous	T8
D16T2	cef	4	T2	2	Multiparous	D16
L11T2	ubro	5	T2	3	Multiparous	L11
D17T1	cef	4	T1	2	Multiparous	D17
D9C	cef	3	T0	1	Primiparous	D9
P20C	cef	3	T0	1	Primiparous	P20

P10T1	cef	3	T1	1	Primiparous	P10
C19T1	noab	2	T1	0	Nulliparous	C19
C3C	noab	3	T0	1	Primiparous	C3
C1T3	noab	3	T3	1	Primiparous	C1
L13T1	ubro	3	T1	1	Primiparous	L13
C7T1	noab	3	T1	1	Primiparous	C7
C5T2	noab	2	T2	0	Nulliparous	C5
C2C	noab	2	T0	0	Nulliparous	C2
C1T1	noab	3	T1	1	Primiparous	C1
T17T2	ubro	3	T2	1	Primiparous	T17
L13C	ubro	3	T0	1	Primiparous	L13
D17T2	cef	4	T2	2	Multiparous	D17
L6C	ubro	3	T0	1	Primiparous	L6
L10T3	ubro	5	T3	3	Multiparous	L10
D13T3	cef	3	T3	1	Primiparous	D13
D13T1	cef	3	T1	1	Primiparous	D13
C3T2	noab	3	T2	1	Primiparous	C3
C19T2	noab	2	T2	0	Nulliparous	C19
P19C	cef	3	T0	1	Primiparous	P19
P19T3	cef	3	T3	1	Primiparous	P19
C1C	noab	3	T0	1	Primiparous	C1
L13T3	ubro	3	T3	1	Primiparous	L13
P9T3	cef	3	T3	1	Primiparous	P9
L10T2	ubro	5	T2	3	Multiparous	L10

P9T2	cef	3	T2	1	Primiparous	P9
C7T2	noab	3	T2	1	Primiparous	C7
D16C	cef	4	T0	2	Multiparous	D16
C4T1	noab	2	T1	0	Nulliparous	C4
T8C	ubro	5	T0	3	Multiparous	T8
P20T2	cef	3	T2	1	Primiparous	P20
C4C	noab	2	T0	0	Nulliparous	C4
P19T1	cef	3	T1	1	Primiparous	P19
C7C	noab	3	T0	1	Primiparous	C7
D9T2	cef	3	T2	1	Primiparous	D9
T8T2	ubro	5	T2	3	Multiparous	T8
C5C	noab	2	T0	0	Nulliparous	C5
L5T3	ubro	3	T3	1	Primiparous	L5
L5T2	ubro	3	T2	1	Primiparous	L5
C2T3	noab	2	T3	0	Nulliparous	C2
T17T1	ubro	3	T1	1	Primiparous	T17
P20T3	cef	3	T3	1	Primiparous	P20
C19T3	noab	2	T3	0	Nulliparous	C19
C12T2	noab	3	T2	1	Primiparous	C12
D9T1	cef	3	T1	1	Primiparous	D9
C4T2	noab	2	T2	0	Nulliparous	C4
D13T2	cef	3	T2	1	Primiparous	D13
D9T3	cef	3	T3	1	Primiparous	D9
C3T3	noab	3	T3	1	Primiparous	C3

C2T2	noab	2	T2	0	Nulliparous	C2
P19T2	cef	3	T2	1	Primiparous	P19

Table S3. Table showing coefficients obtained from differential abundance analysis using Songbird tool with formula C (Group, Treatment('NOAB')).

featureid	Intercept	C(Group2, Treatment('noab'))[T.cef]	C(Group2, Treatment('noab'))[T.ubro]
k__Bacteria|p__Proteobacteria|c__Gammaproteobacteria|o__Pseudomonadales|f__Moraxellaceae|g__*Psychrobacter*	-0.48714	-0.49549	-0.49071
k__Bacteria|p__Proteobacteria|c__Gammaproteobacteria|o__Pseudomonadales|f__Moraxellaceae|g__*Acinetobacter*	0.104539	0.021822	0.20377
k__Bacteria|p__Proteobacteria|c__Gammaproteobacteria|o__Pseudomonadales|f__Moraxellaceae|g__*Moraxella*	-0.18873	-0.06052	-0.03973
k__Bacteria|p__Proteobacteria|c__Gammaproteobacteria|o__Pseudomonadales|f__Pseudomonadaceae|g__*Pseudomonas*	0.822938	0.23601	0.118122
k__Bacteria|p__Proteobacteria|c__Gammaproteobacteria|o__Enterobacterales|f__Enterobacteriaceae|g__	-0.22715	-0.06166	-0.05006

k__Bacteria|p__Proteobacteria|c__Gammaproteobacteria|o__Enterobacterales|f__Enterobacteriaceae|g__*Escherichia*	-0.21432	-0.06126	-0.04628
k__Bacteria|p__Proteobacteria|c__Gammaproteobacteria|o__Enterobacterales|f__Yersiniaceae|g__*Serratia*	-0.2413	-0.06436	-0.05548
k__Bacteria|p__Proteobacteria|c__Gammaproteobacteria|o__Enterobacterales|f__Erwiniaceae|g__*Buchnera*	-0.21317	-0.06081	-0.04642
k__Bacteria|p__Proteobacteria|c__Gammaproteobacteria|o__Enterobacterales|f__Pectobacteriaceae|g__*Sodalis*	-0.21525	-0.05879	-0.04835
k__Bacteria|p__Proteobacteria|c__Gammaproteobacteria|o__Xanthomonadales|f__Xanthomonadaceae|g__	-0.20194	-0.05346	-0.04568
k__Bacteria|p__Proteobacteria|c__Gammaproteobacteria|o__Xanthomonadales|f__Xanthomonadaceae|g__*Stenotrophomonas*	0.340684	0.166177	0.065002
k__Bacteria|p__Proteobacteria|c__Gammaproteobacteria|o__Xanthomonadales|f__Xanthomonadaceae|g__*Lysobacter*	0.259635	0.25292	-0.01833
k__Bacteria|p__Proteobacteria|c__Gammaproteobacteria|o__Methylococcales|f__Methylococcaceae|g__*Methylovulum*	-0.21223	-0.0552	-0.03209
k__Bacteria|p__Proteobacteria|c__Alphaproteobacteria|o__Rhodobacterales|f__Rhodobacteraceae|g__	-0.19415	-0.06025	-0.02359

k__Bacteria|p__Proteobacteria|c__Alphaproteobacteria|o__Rhodobacterales|f__Rhodobacteraceae|g__*Paracoccus*	-0.11266	-0.05799	0.06665
k__Bacteria|p__Proteobacteria|c__Alphaproteobacteria|o__Rhodobacterales|f__Rhodobacteraceae|g__*Rhodobacter*	-0.21346	-0.06027	-0.03847
k__Bacteria|p__Proteobacteria|c__Alphaproteobacteria|o__Rhizobiales|f__|g__	-0.15449	-0.02776	-0.02639
k__Bacteria|p__Proteobacteria|c__Alphaproteobacteria|o__Rhizobiales|f__Phyllobacteriaceae|g__*Mesorhizobium*	-0.19888	-0.04567	-0.04165
k__Bacteria|p__Proteobacteria|c__Alphaproteobacteria|o__Rhizobiales|f__Phyllobacteriaceae|g__*Aminobacter*	-0.20662	-0.04857	-0.04445
k__Bacteria|p__Proteobacteria|c__Alphaproteobacteria|o__Rhizobiales|f__Phyllobacteriaceae|g__*Phyllobacterium*	-0.21616	-0.04196	-0.05048
k__Bacteria|p__Proteobacteria|c__Alphaproteobacteria|o__Rhizobiales|f__Bradyrhizobiaceae|g__*Rhodopseudomonas*	-0.2002	-0.04949	-0.04099
k__Bacteria|p__Proteobacteria|c__Alphaproteobacteria|o__Rhizobiales|f__Brucellaceae|g__*Ochrobactrum*	-0.18801	-0.0127	-0.05095
k__Bacteria|p__Proteobacteria|c__Alphaproteobacteria|o__Caulobacterales|f__Caulobacteraceae|g__*Brevundimonas*	-0.21691	-0.05234	-0.04544

k__Bacteria|p__Proteobacteria|c__Betaproteobacteria|o__Burkholderiales|f__|g__	-0.17826	-0.03499	-0.0341
k__Bacteria|p__Proteobacteria|c__Betaproteobacteria|o__Burkholderiales|f__Comamonadaceae|g__	-0.16555	-0.01551	-0.03083
k__Bacteria|p__Proteobacteria|c__Betaproteobacteria|o__Burkholderiales|f__Comamonadaceae|g__*Comamonas*	-0.20775	-0.04027	-0.04723
k__Bacteria|p__Proteobacteria|c__Betaproteobacteria|o__Burkholderiales|f__Comamonadaceae|g__*Variovorax*	-0.1943	-0.05054	-0.03201
k__Bacteria|p__Proteobacteria|c__Betaproteobacteria|o__Burkholderiales|f__Comamonadaceae|g__*Delftia*	-0.01807	0.130668	-0.03337
k__Bacteria|p__Proteobacteria|c__Betaproteobacteria|o__Burkholderiales|f__Comamonadaceae|g__*Ottowia*	0.187391	0.303989	0.04468
k__Bacteria|p__Proteobacteria|c__Betaproteobacteria|o__Burkholderiales|f__Alcaligenaceae|g__*Alcaligenes*	-0.09802	0.096201	-0.06153
k__Bacteria|p__Proteobacteria|c__Betaproteobacteria|o__Burkholderiales|f__Burkholderiaceae|g__*Polynucleobacter*	0.202905	0.07999	0.046725
k__Bacteria|p__Proteobacteria|c__Betaproteobacteria|o__Burkholderiales|f__Oxalobacteraceae|g__*Janthinobacterium*	-0.20971	-0.03783	-0.05259
k__Bacteria|p__Actinobacteria|c__Actinobacteria|o__Micrococcales|f__|g__	-0.02247	-0.01838	0.018418

k__Bacteria|p__Actinobacteria|c__Actinobacteria|o__Micrococcales|f__Micrococcaceae|g__	-0.13686	-0.04669	-0.02187
k__Bacteria|p__Actinobacteria|c__Actinobacteria|o__Micrococcales|f__Micrococcaceae|g__*Arthrobacter*	0.206154	-0.02504	0.030991
k__Bacteria|p__Actinobacteria|c__Actinobacteria|o__Micrococcales|f__Micrococcaceae|g__*Glutamicibacter*	-0.14841	-0.05916	-0.02827
k__Bacteria|p__Actinobacteria|c__Actinobacteria|o__Micrococcales|f__Micrococcaceae|g__*Kocuria*	0.727719	0.393234	0.473597
k__Bacteria|p__Actinobacteria|c__Actinobacteria|o__Micrococcales|f__Micrococcaceae|g__*Rothia*	-0.21063	-0.06299	-0.04428
k__Bacteria|p__Actinobacteria|c__Actinobacteria|o__Micrococcales|f__Micrococcaceae|g__*Micrococcus*	-0.13219	-0.05269	0.026983
k__Bacteria|p__Actinobacteria|c__Actinobacteria|o__Micrococcales|f__Micrococcaceae|g__*Sinomonas*	-0.2013	-0.05532	-0.03988
k__Bacteria|p__Actinobacteria|c__Actinobacteria|o__Micrococcales|f__Microbacteriaceae|g__	-0.16104	-0.04075	-0.02757
k__Bacteria|p__Actinobacteria|c__Actinobacteria|o__Micrococcales|f__Microbacteriaceae|g__*Microbacterium*	0.878039	0.503077	0.209511
k__Bacteria|p__Actinobacteria|c__Actinobacteria|o__Micrococcales|f__Microbacteriaceae|g__*Agromyces*	-0.20839	-0.05587	-0.04294
k__Bacteria|p__Actinobacteria|c__Actinobacteria|o__Micrococcales|f__Microbacteriaceae|g__*Clavibacter*	-0.20502	-0.05873	-0.04458

k__Bacteria|p__Actinobacteria|c__Actinobacteria|o__Micrococcales|f__Microbacteriaceae|g__*Leifsonia*	-0.19225	-0.05052	-0.03915
k__Bacteria|p__Actinobacteria|c__Actinobacteria|o__Micrococcales|f__Microbacteriaceae|g__*Microterricola*	-0.21058	-0.05393	-0.04648
k__Bacteria|p__Actinobacteria|c__Actinobacteria|o__Micrococcales|f__Microbacteriaceae|g__*Frondihabitans*	-0.21036	-0.05969	-0.04549
k__Bacteria|p__Actinobacteria|c__Actinobacteria|o__Micrococcales|f__Dermabacteraceae|g__*Brachybacterium*	1.100255	-0.10812	-0.01632
k__Bacteria|p__Actinobacteria|c__Actinobacteria|o__Micrococcales|f__Intrasporangiaceae|g__*Janibacter*	-0.1361	-0.05692	0.026611
k__Bacteria|p__Actinobacteria|c__Actinobacteria|o__Micrococcales|f__Intrasporangiaceae|g__*Intrasporangium*	-0.18586	-0.05871	-0.03145
k__Bacteria|p__Actinobacteria|c__Actinobacteria|o__Micrococcales|f__Intrasporangiaceae|g__*Serinicoccus*	-0.18811	-0.05803	-0.02364
k__Bacteria|p__Actinobacteria|c__Actinobacteria|o__Micrococcales|f__Intrasporangiaceae|g__*Arsenicicoccus*	-0.18527	-0.05417	-0.02229
k__Bacteria|p__Actinobacteria|c__Actinobacteria|o__Micrococcales|f__Brevibacteriaceae|g__*Brevibacterium*	1.510723	-0.26926	-0.25562

k__Bacteria|p__Actinobacteria|c__Actinobacteria|o__Micrococcales|f__Dermacoccaceae|g__*Luteipulveratus*	-0.2025	-0.05754	-0.03512
k__Bacteria|p__Actinobacteria|c__Actinobacteria|o__Micrococcales|f__Dermacoccaceae|g__*Kytococcus*	-0.20443	-0.05523	-0.03523
k__Bacteria|p__Actinobacteria|c__Actinobacteria|o__Micrococcales|f__Dermacoccaceae|g__*Dermacoccus*	-0.19912	-0.05235	-0.03468
k__Bacteria|p__Actinobacteria|c__Actinobacteria|o__Micrococcales|f__Beutenbergiaceae|g__*Beutenbergia*	-0.21159	-0.0552	-0.04207
k__Bacteria|p__Actinobacteria|c__Actinobacteria|o__Micrococcales|f__Beutenbergiaceae|g__*Miniimonas*	-0.19863	-0.05425	-0.0283
k__Bacteria|p__Actinobacteria|c__Actinobacteria|o__Micrococcales|f__Sanguibacteraceae|g__*Sanguibacter*	-0.20739	-0.0561	-0.0427
k__Bacteria|p__Actinobacteria|c__Actinobacteria|o__Corynebacteriales|f__|g__	-0.10821	-0.01763	-0.00379
k__Bacteria|p__Actinobacteria|c__Actinobacteria|o__Corynebacteriales|f__Corynebacteriaceae|g__*Corynebacterium*	1.661905	-0.00768	0.438415
k__Bacteria|p__Actinobacteria|c__Actinobacteria|o__Corynebacteriales|f__Nocardiaceae|g__*Rhodococcus*	1.499408	0.988738	0.605951
k__Bacteria|p__Actinobacteria|c__Actinobacteria|o__Corynebacteriales|f__Dietziaceae|g__*Dietzia*	-0.11913	-0.04926	0.010988
k__Bacteria|p__Actinobacteria|c__Actinobacteria|o__Corynebacteriales|f__Mycobacteriaceae|g__	-0.1052	0.019298	-0.01502

k__Bacteria|p__Actinobacteria|c__Actinobacteria|o__Corynebacteriales|f__Mycobacteriaceae|g__*Mycobacterium*	-0.07432	0.0247	-0.00822
k__Bacteria|p__Actinobacteria|c__Actinobacteria|o__Corynebacteriales|f__Mycobacteriaceae|g__*Mycobacteroides*	0.210905	0.297149	0.065613
k__Bacteria|p__Actinobacteria|c__Actinobacteria|o__Corynebacteriales|f__Tsukamurellaceae|g__*Tsukamurella*	-0.17258	-0.01866	-0.04098
k__Bacteria|p__Actinobacteria|c__Actinobacteria|o__Streptomycetales|f__Streptomycetaceae|g__*Streptomyces*	0.04393	0.025371	0.039756
k__Bacteria|p__Actinobacteria|c__Actinobacteria|o__Pseudonocardiales|f__Pseudonocardiaceae|g__*Actinoalloteichus*	3.228573	0.130644	0.527985
k__Bacteria|p__Actinobacteria|c__Actinobacteria|o__Pseudonocardiales|f__Pseudonocardiaceae|g__*Pseudonocardia*	-0.20553	-0.05877	-0.03884
k__Bacteria|p__Actinobacteria|c__Actinobacteria|o__Propionibacteriales|f__Nocardioidaceae|g__*Nocardioides*	-0.08152	-0.03465	0.019309
k__Bacteria|p__Actinobacteria|c__Actinobacteria|o__Propionibacteriales|f__Nocardioidaceae|g__*Aeromicrobium*	-0.20082	-0.05963	-0.04363

k__Bacteria|p__Actinobacteria|c__Actinobacteria|o__ Propionibacteriales|f__Nocardioidaceae|g__*Pimelobacter*	-0.18516	-0.05011	-0.03011
k__Bacteria|p__Actinobacteria|c__Actinobacteria|o__ Propionibacteriales|f__Nocardioidaceae|g__*Kribbella*	-0.21425	-0.05734	-0.04205
k__Bacteria|p__Actinobacteria|c__Actinobacteria|o__ Propionibacteriales|f__Nocardioidaceae|g__*Micropruina*	-0.1825	-0.04948	-0.02635
k__Bacteria|p__Actinobacteria|c__Actinobacteria|o__ Propionibacteriales|f__Propionibacteriaceae|g__	-0.17764	-0.02195	-0.03498
k__Bacteria|p__Actinobacteria|c__Actinobacteria|o__ Propionibacteriales|f__Propionibacteriaceae|g__*Tessaracoccus*	-0.05011	-0.00429	0.034666
k__Bacteria|p__Actinobacteria|c__Actinobacteria|o__ Propionibacteriales|f__Propionibacteriaceae|g__*Acidipropionibacterium*	0.096559	0.175055	0.057006
k__Bacteria|p__Actinobacteria|c__Actinobacteria|o__ Propionibacteriales|f__Propionibacteriaceae|g__*Cutibacterium*	-0.15491	-0.03241	-0.03512
k__Bacteria|p__Actinobacteria|c__Actinobacteria|o__ Propionibacteriales|f__Propionibacteriaceae|g__*Propionibacterium*	-0.0024	0.079033	0.044894
k__Bacteria|p__Actinobacteria|c__Actinobacteria|o__ Propionibacteriales|f__Propionibacteriaceae|g__*Microlunatus*	-0.18241	-0.05756	-0.01455

k__Bacteria|p__Actinobacteria|c__Actinobacteria|o__Micromonosporales|f__Micromonosporaceae|g__*Plantactinospora*	0.278867	0.040809	0.097554
k__Bacteria|p__Actinobacteria|c__Actinobacteria|o__Bifidobacteriales|f__Bifidobacteriaceae|g__*Bifidobacterium*	0.103772	0.083442	0.033794
k__Bacteria|p__Actinobacteria|c__Actinobacteria|o__Nakamurellales|f__Nakamurellaceae|g__*Nakamurella*	-0.19973	-0.04952	-0.03668
k__Bacteria|p__Actinobacteria|c__Coriobacteriia|o__Eggerthellales|f__Eggerthellaceae|g__*Gordonibacter*	-0.0631	-0.07634	-0.06444
k__Bacteria|p__Actinobacteria|c__Thermoleophilia|o__Solirubrobacterales|f__Conexibacteraceae|g__*Conexibacter*	-0.1998	-0.03787	-0.04831
k__Bacteria|p__Firmicutes|c__Bacilli|o__Lactobacillales|f__|g__	-0.20243	-0.04009	-0.04365
k__Bacteria|p__Firmicutes|c__Bacilli|o__Lactobacillales|f__Streptococcaceae|g__*Streptococcus*	-0.17518	-0.00236	-0.04769
k__Bacteria|p__Firmicutes|c__Bacilli|o__Lactobacillales|f__Streptococcaceae|g__*Lactococcus*	-0.06575	0.08604	-0.02159
k__Bacteria|p__Firmicutes|c__Bacilli|o__Lactobacillales|f__Lactobacillaceae|g__	-0.22052	-0.04908	-0.04804
k__Bacteria|p__Firmicutes|c__Bacilli|o__Lactobacillales|f__Lactobacillaceae|g__*Lactobacillus*	0.155053	0.21733	0.090622
k__Bacteria|p__Firmicutes|c__Bacilli|o__Lactobacillales|f__Enterococcaceae|g__*Enterococcus*	-0.20221	-0.04571	-0.03201

k__Bacteria|p__Firmicutes|c__Bacilli|o__Lactobacillales|f__Leuconostocaceae|g__*Leuconostoc*	-0.17699	-0.02222	-0.03086
k__Bacteria|p__Firmicutes|c__Bacilli|o__Bacillales|f__Staphylococcaceae|g__*Staphylococcus*	0.241502	-0.05603	-0.02029
k__Bacteria|p__Firmicutes|c__Clostridia|o__Clostridiales|f__Clostridiaceae|g__*Clostridium*	-0.14928	-0.0416	-0.03513
k__Bacteria|p__Firmicutes|c__Negativicutes|o__Veillonellales|f__Veillonellaceae|g__*Negativicoccus*	0.166914	0.013254	0.064588
k__Bacteria|p__Deinococcus-Thermus|c__Deinococci|o__Deinococcales|f__Deinococcaceae|g__*Deinococcus*	-0.21875	-0.06637	-0.02945
k__Bacteria|p__Tenericutes|c__Mollicutes|o__Mycoplasmatales|f__Mycoplasmataceae|g__*Mycoplasma*	-0.20723	-0.05714	-0.04786
k__Bacteria|p__Bacteroidetes|c__Sphingobacteriia|o__Sphingobacteriales|f__Sphingobacteriaceae|g__*Sphingobacterium*	-0.22744	-0.06315	-0.05452
k__Bacteria|p__Bacteroidetes|c__Flavobacteriia|o__Flavobacteriales|f__Flavobacteriaceae|g__*Chryseobacterium*	-0.16978	0.000731	-0.0474
k__Bacteria|p__Verrucomicrobia|c__|o__|f__|g__	-0.20056	-0.05548	-0.04549
